# Cognitive frailty and functional disability in older adults: A 10-year prospective cohort study in Japan

**DOI:** 10.1007/s11357-024-01461-0

**Published:** 2024-12-03

**Authors:** Sanmei Chen, Tao Chen, Takanori Honda, Hiro Kishimoto, Yu Nofuji, Kenji Narazaki

**Affiliations:** 1 Graduate School of Biomedical and Health Sciences, Hiroshima University, Minami Ward, 1-2-3 Kasumi, Hiroshima, 734-8551 Japan; 2 Sports and Health Research Center, Department of Physical Education, Tongji University, 1239 Siping Road, Shanghai, 200-092 China; 3 Center for Cohort Studies, Graduate School of Medical Sciences, Kyushu University, 3-1-1 Maidashi, Higashi-Ku, Fukuoka, 812-8582 Japan; 4 Faculty of Arts and Science, Kyushu University, 744 Motooka Nishi-Ku, Fukuoka, 819-0395 Japan; 5 Research Team for Social Participation and Healthy Aging, Tokyo Metropolitan Institute for Geriatrics and Gerontology, 35-2 Sakae, Itabashi, Tokyo 173-0015 Japan; 6 Center for Liberal Arts, Fukuoka Institute of Technology, 3-30-1 Wajiro-Higashi, Higashi-Ku, Fukuoka, 811-0295 Japan

**Keywords:** Cognitive frailty, Accelerometry, Long-term care needs, Risk factors

## Abstract

Cognitive frailty is considered a clinical entity associated with a high risk for adverse health outcomes. However, its clinical significance remains poorly understood. This study investigated the association between cognitive frailty and risk for functional disability among community-dwelling older adults. In total, 1,597 Japanese adults aged ≥ 65 years that were free of dementia and functional disability at baseline were prospectively followed for 10 years. Cognitive frailty was defined as the presence of both physical frailty (using Cardiovascular Health Study criteria) and cognitive impairment (Mini-Mental State Examination score < 26 points). Functional disability was identified using Japan’s Long-term Care Insurance System database. Cox proportional hazard models were used to estimate the hazard ratios (HR) for cognitive frailty on the risk for functional disability. Functional disability was identified in 488 participants during follow-up. A multiplicative interaction effect between cognitive impairment and physical frailty on the risk of disability was observed (*p* = 0.045). Compared with the robust group, the multivariable-adjusted HRs (95% confidence interval) for functional disability were 3.70 (2.37‒5.77) for cognitive frailty, 2.51 (1.81‒3.47) for physical pre-frailty with cognitive impairment, 2.16 (1.42‒3.29) for cognitive impairment only, 1.95 (1.36‒2.80) for physical frailty only, and 1.94 (1.53‒2.46) for physical pre-frailty only. These associations remained after adjusting for the competing risk for death and in sensitivity analyses to minimize potential reverse causality. Cognitive frailty is associated with an increased risk for functional disability in community-dwelling older adults. Cognitive frailty may be a clinically important target for the prevention of functional disability.

**Supplementary Information:**

The online version contains supplementary material available at 10.1007/s11357-024-01461-0.

## Introduction

Over 14% of older adults worldwide are unable to meet their basic needs due to limited functional ability [1]. In Japan, the number of individuals aged 65 and over with Long-term Care Insurance (LTCI) certification for functional disability rose from 2.18 million in 2000 to 6.69 million in 2020 [2]. Functional disability in older adults, such as dependence in activities of daily living (ADL) and instrumental ADL (IADL), results in increased use of acute care [3], hospitalization [4], and elevated mortality risk [5]. It imposes significant burdens on social structures, economic resources, and healthcare systems [6, 7]. Identifying risk factors for functional disability is urgently needed to inform public health recommendations and preventive strategies in response to aging populations.

Cognitive frailty is a clinical condition whereby aging affects both physical and cognitive functioning, and has been proposed as a distinctive entity [8]. Cognitive frailty is defined by concurrent physical frailty and cognitive without overt dementia diagnosis [8]. This concept arises from the recognized connection between physical frailty and cognitive decline, both sharing common pathophysiological mechanisms and health outcomes [9–14]. A cohort study in America found that including cognitive measures alongside physical frailty significantly improved the ability to predict adverse outcomes, such as ADL disability [15]. This indicated the importance of assessing both physical frailty and cognitive function when assessing the risk of functional disability [15]. Despite this, the clinical significance of cognitive frailty concerning functional disability is not yet fully understood [16, 17].

While existing literature suggests that cognitive frailty could be a key target for preventing functional disability, potentially more so than physical frailty or cognitive impairment alone [13]. However, empirical evidence quantifying this association remains limited [17]. Cross-sectional studies have shown that individuals with cognitive frailty are more likely to experience functional disability compared to those without, but such studies are constrained by their design [18–20]. Only a few prospective studies have explored the extent to which cognitive frailty increased the risk for incident functional disability among older adults [15, 16]. Four longitudinal studies found increased odds of disability associated with cognitive frailty at baseline [21–24]. However, those studies lacked time-to-event data to estimate hazard ratios (HR) for incident functional disability [21–24]. One Japanese cohort study with time-to-event data found that cognitive frailty was associated with a high risk for functional disability; but its short (2-year) follow-up period raised concerns about potential reverse causation bias [25].

This study used time-to-event data from a prospective cohort study of community-dwelling older adults in Japan to investigate the association between baseline cognitive frailty and the subsequent risk of incident functional disability over a ten-year follow-up period. We hypothesised that cognitive impairment and physical frailty would have a significant interaction effect on the risk of functional disability, and that cognitive frailty would be associated with an elevated risk of functional disability.

## Methods

### Participants

The Sasaguri Genkimon Study (SGS) is an ongoing community-based prospective study started in 2011, in Sasaguri town, a suburb in Fukuoka Prefecture, Japan. This study aimed to explore risk and protective factors related to long-term care needs [26–28]. The inclusion criteria of the SGS included: 1) residents of the Sasaguri Town; 2) aged ≥ 65 years; 3) not being identified as having functional disability in the national LTCI at baseline as of January 2011. In Japan, LTCI is a mandatory social insurance system for benefits strictly based on physical and mental disability [30].

As of January 2011, 4,979 residents met the SGS criteria. We contacted 4,913 eligible residents, as 66 had died or moved away during the baseline survey period (May to August 2011). A total of 53.5% agreed to participate (n = 2,629). For the present study, we excluded participants certified as requiring LTCI before the baseline (n = 9), having a medical history of dementia (n = 10), Parkinson’s disease (a progressive neurodegenerative condition that could possibly quality for LTCI) (n = 3), or had Mini-Mental State Examination (MMSE) scores < 18, indicative of probable dementia (n = 12). This left 2,593 eligible participants. Further exclusions included 780 participants without complete data for physical frailty measures, 144 without MMSE data, and 72 with incomplete covariate data, resulting in an analytic cohort of 1,597 participants (see Supplemental Fig.1). Supplemental Table 1 shows the characteristics of both included and excluded participants. This study was conducted in accordance with the Declaration of Helsinki and approved by the Institutional Review Board of Fukuoka Institute of Technology, Japan. We obtained written informed consent from all participants.

### Follow-up survey of incident functional disability

Participants were prospectively followed from the baseline survey until functional disability was ascertained, loss to follow-up because of moving away from town, or the end of follow-up (March 31, 2021), whichever came first. Data were provided by the Sasaguri Municipal Government Office. Information on participants’ death or moving away from the town during follow-up was drawn from the resident registration system. Newly-onset functional disability was ascertained using the nationally uniform LTCI system database. In this system, certification of functional disability was determined based on nationally standardized assessments of an individual’s physical and mental status. These standardized assessments included paralysis and limitation of joint movement, movement and balance, complex movement, conditions requiring special assistance, conditions requiring assistance with ADL/IADL, communication and cognition, and behavioral problems. Specifically, a computer-aided standardized scoring system is applied based on these standardized assessments of physical and mental function. The amount of time required for care is then estimated across eight categories: grooming and bathing, eating, using the toilet, transferring, assistance with IADL, behavioral problems, rehabilitation, and medical services. Next, a local certification committee comprising physicians, nurses, and other health and social services experts decides whether a given adult should be certified or not. Finally, the committee assigns care needs at one of seven levels (support level 1–2; care level 1–5) to each certified person. The LTCI care needs levels have been shown to be highly correlated with physical disability levels as identified by the Barthel Index (Spearman’s ρ = –0.86) [30]. We defined the endpoint (i.e., functional disability) as the onset of long-term care needs at the first level (support level 1) or above [28, 30, 30, 30].

### Cognitive frailty measures at baseline

Cognitive-frailty was defined as the simultaneous presence of physical frailty with cognitive impairment without concurrent dementia [8]. Participants were categorized into six groups: robust (physically non-frail and cognitively normal), physical pre-frailty only (physically pre-frail and cognitively normal), physical frailty only (physically frail and cognitively normal), cognitive impairment only (physically non-frail and cognitively impaired), physical pre-frailty with cognitive impairment (physically pre-frail and cognitively impaired), and cognitive frailty (physically frail and cognitively impaired).

Based on the original definition [8], physical frailty was identified using the five Cardiovascular Health Study frailty phenotype criteria: weight loss, exhaustion, low grip strength, slow gait speed, and low physical activity [30]. Participants meeting three or more criteria were considered physically frail, those one or two criteria as physically pre-frail, and those with none as physically robust. The cutoff points for low grip strength, slow gait speed, and low physical activity were determined using the population-based lowest quintile approach, classifying participants in the lowest quintile as affected [30, 30]. Weight loss was defined as self-reported unintentional weight loss of more than 2–3 kg in the previous 6 months [30]. Exhaustion was indicated by a positive answer to one (or both) of two questions: “Did you feel that everything you did was an effort?” and “Did you feel exhausted without any reason?” in the last month. Grip strength (kg) was measured using a handhold dynamometer (GRIP-D, T.K.K. 5401, Takei Scientific Instruments Co. Ltd., Niigata, Japan), with the lowest quintile cutoff stratified by sex and body mass index (BMI). Gait speed was measured over a 5-m walking test at the participant’s maximum walking speed, with the lowest quintile cutoff stratified by sex and standing height. Physical activity was objectively assessed using a tri-axial accelerometer (Active Style Pro, HJA350-IT, Omron Healthcare, Inc., Kyoto, Japan) and presented as kcal/kg (body weight) per day, with the lowest quintile cutoff stratified by sex. Participants wore the accelerometer on their waist for seven consecutive days during waking hours and only removed it for water-based activities. This operational definition of physical frailty was previously reported to have satisfactory internal validity for this population [30].

Cognitive function was measured using the Japanese version of the MMSE [30]. The MMSE is considered appropriate and feasible to measure cognitive performance for screening cognitive frailty in primary care settings [8, 17, 20]. MMSE scores range from 0 to 30 points, with higher scores indicating better global cognitive function. Cognitive impairment was defined as an MMSE score < 26, a commonly used cutoff point for the cognitive measure of cognitive frailty [17].

### Covariate measures at baseline

Information on participants’ age and sex was obtained from the Sasaguri Municipality Office. Years of formal education, currently employed (yes or no), living alone (yes or no), economic status (comfortable/relatively comfortable or relatively uncomfortable/uncomfortable), currently smoking (yes or no), currently drinking (yes or no), habitual exercise (yes or no), and experienced a fall in the previous year (yes or no) were measured using a questionnaire. Body weight and height were measured using conventional scales. BMI was calculated by dividing the body mass (kg) by height in meters squared (kg/m^2^). Medical history of chronic diseases was self-reported in the questionnaire, in which participants indicated whether they had been diagnosed with any of these conditions by a physician. Multimorbidity was defined as the presence of two or more of 13 listed chronic diseases: hypertension, stroke, chronic heart disease, diabetes mellitus, hyperlipidemia, respiratory disease, digestive disease, kidney disease, osteoarthritis or rheumatism, trauma fracture, cancer, ear disease, and eye disease. The selection of covariates was based on literature and the principles of confounder selection by TJ. Vander Weele [30].

### Statistical analyses

Participants’ characteristics at baseline were summarized using means (standard deviation [SD]), medians (interquartile range [IQR]), or proportions across the six cognitive frailty groups, as appropriate. Pairwise comparisons with the robust group were adjusted for multiple comparisons using Dunnett’s test with analysis of variance (ANOVA) for continuous variables and logistic regression for categorical variables.

The incidence rate of functional disability was calculated using the person-year method. Cumulative survival rates of functional disability for the six cognitive frailty groups were illustrated with Kaplan–Meier curves. The survival curves were compared with a log-rank test. Cox proportional hazard models were used to estimate the HR and 95% confidence interval (CI) for functional disability based on cognitive frailty status at baseline. A crude model was constructed, followed by adjustments for sex and age. Next, a multivariable-adjusted model was developed with additional adjustment for education, economic status, current employment, living alone, smoking, drinking, exercise habit, BMI, history of falls, multimorbidity, history of hypertension (yes or no), stroke (yes or no), chronic heart disease (yes or no), and diabetes mellitus (yes or no). In a prior step, we estimated the HR and 95% CI for functional disability based on baseline cognitive impairment and physical frailty status. We also tested the multiplicative interaction effect between cognitive impairment (yes or no) and physical frailty status (non-frail, pre-frail, or frail) in the multivariable-adjusted model.

Standard Cox proportional hazard models may overestimate the event risk due to censoring participants who died before the event, particularly in studies among older adults [30]. We repeated the analysis using the Fine and Grey sub-distribution hazard model to account for competing risks of death [30]. We tested the interactions of sex and age groups (< 75 years and ≥ 75 years) with cognitive frailty to examine potential moderating effects.

Finally, we performed sensitivity analyses by excluding participants with a self-reported history of depression who could have exhibited cognitive frailty because of this single condition (n = 12) and participants with MMSE score < 21 at baseline to consider the chance of probable dementia (n = 13). To account for potential reverse-causation, we conducted the two sensitivity analyses by excluding: 1) participants certified as having functional disability in the first 2 years of follow-up (n = 59); and 2) participants aged ≥ 85 years (n = 78). All tests were two-tailed and interpreted at a significance level of α = 0.05. All statistical analyses were conducted using SAS version 9.4 (SAS Institute Inc., Cary, NC).

## Results

Participants’ mean age at baseline was 73.3 (SD 6.0) years and 39.1% were men.

Overall, 2.9% of participants were classified as cognitive frailty, 8.6% as physical pre-frailty with cognitive impairment, 5.4% as cognitive impairment only, 6.4% as physical frailty only, and 35.6% as physical pre-frailty only. Table 1 shows participants’ characteristics by baseline cognitive frailty status. Compared with the robust group, participants in the cognitive frailty group were more likely to be older, have few years of education, a history of falls, multimorbidity, and a lower level of MMSE score. They were also less likely to be currently employed or drinking at baseline. Additionally, participants in cognitive frailty group had a significant lower level of MMSE score than those in the cognitive impairment only group (*p* < 0.001).

**Table 1:** Baseline characteristics of study participants by cognitive frailty status (N = 1,597)

	Robust	Physical pre-frailty only	Physical frailty only	Cognitive impairment only	Physical pre-frailty and cognitive impairment	Cognitive frailty
	(n = 657)	(n = 568)	(n = 103)	(n = 86)	(n = 137)	(n = 46)
Age, years	71.4 (5.0)	73.6 (5.8)^*^	78.4 (5.9)^*^	72.6 (5.4)	75.5 (6.2)^*^	81.3 (6.2)^*^
Men, %	37.8	40.3	37.9	50.0	34.3	41.3
Education, years	11.7 (2.4)	11.1 (2.4)^*^	10.3 (2.1)^*^	10.1 (2.4)^*^	9.6 (2.2)^*^	9.8 (2.4)^*^
Economic status, very uncomfortable or uncomfortable, %	41.1	39.8	38.8	40.7	32.1	41.3
Currently employed, %	22.2	16.9	6.8^*^	8.1^*^	12.4	4.4^*^
Living alone, %	12.0	16.7	17.5	2.3	11.0	10.9
Currently smoking, %	6.7	8.6	7.8	15.1^*^	5.8	4.3
Currently drinking, %	42.5	39.8	28.2^*^	40.7	33.6	23.9^*^
Having exercise habit, %	66.2	58.5^*^	60.2	57.0	56.2	58.7
BMI, kg/m^2^	22.9 (2.8)	23.5 (3.2)^*^	23.5 (3.3)	22.9 (3.3)	23.4 (3.8)	23.1 (4.2)
Having a history of fall, %	13.9	23.2^*^	33.0^*^	8.1	24.1^*^	39.1^*^
Multimorbidity, %^†^	39.9	50.0^*^	77.7^*^	43.0	44.5	69.6^*^
History of hypertension, %	33.0	41.2^*^	40.8	40.7	44.5^*^	47.8^*^
History of stroke, %	2.3	3.0	6.8	2.3	7.3	8.7^*^
History of chronic heart disease, %	10.1	14.1	31.1^*^	11.6	16.8	23.9^*^
History of diabetes mellitus, %	10.5	14.4	21.4^*^	10.5	15.3	19.6
MMSE score, points	28.5 (1.3)	28.4 (1.2)	28.0 (1.4)^*^	24.0 (1.3)^*^	23.8 (1.3)^*^	23.3 (1.9)^*^

Note: Data were presented as mean (standard deviation) or proportion. BMI = body mass index; MMSE = Mini-Mental State Examination

^*^P <0.05. Statistical significance was based on the pairwise comparison versus the robust group using the Dunnett’s test

^†^Multimorbidity was defined as two or more of the listed chronic diseases: hypertension, stroke, chronic heart disease, diabetes mellitus, dyslipidemia, respiratory disease, digestive disease, kidney disease, osteoarthritis or rheumatism, trauma fracture, cancer, ear disease, and eye disease

Dring a median follow-up of 7.5 (IQR 5.3‒9.8) years and 12,491 person-years, 488 participants developed functional disability, 143 died and 58 moved away from town before being ascertained as functional disability. Table 2 shows that both cognitive impairment (HR [95% CI]: 1.57 [1.26‒1.94] vs. cognitively unimpaired) and physical frailty (HR [95% CI]: 1.74 [1.41‒2.16] for being pre-frail and 1.97 [1.45‒2.68] for being frail vs. non-frail) were associated with an increased risk of functional disability. The interaction test revealed a significant multiplicative interaction between cognitive impairment and physical frailty on the risk of functional disability (*p* for interaction = 0.045), whereas no significant interaction was found with physical pre-frailty (*p* for interaction = 0.68).

**Table 2:** Interaction effect between cognitive impairment and physical frailty at baseline on the risk of functional disability over 10 years (n = 1,597)

	HR (95% CI)^*^
Cognitively unimpaired	1.00 (reference)
Cognitively impaired	1.57 (1.26‒1.94)
Physical non-frail	1.00 (reference)
Physical pre-frailty	1.74 (1.41‒2.16)
Physical frailty	1.97 (1.45‒2.68)
^†^*P* for interaction between cognitive impairment and physical pre-frailty	0.68
^†^*P* for interaction between cognitive impairment and physical frailty	0.046

Note: CI = confidence interval; HR = hazard ratio

^*^Adjusted for age (years, continuous), sex (men or women), education (years, continuous), economic status (very uncomfortable/uncomfortable or comfortable/very comfortable), currently employed (yes or no), living alone (yes or no), smoking (yes or no), drinking (yes or no), exercise habit (yes or no), body mass index (kg/m^2^, continuous), history of falls (yes or no), multimorbidity (yes or no), history of hypertension (yes or no), stroke (yes or no), chronic heart disease (yes or no), and diabetes mellitus (yes or no). In addition, cognitive impairment and physical frailty were mutually adjusted

^†^The *p* value for the multiplicative interaction between cognitive impairment (yes or no) and physical frailty status (non-frail, pre-frail, or frail) was calculated in the multivariable-adjusted model

**Fig. 1:**
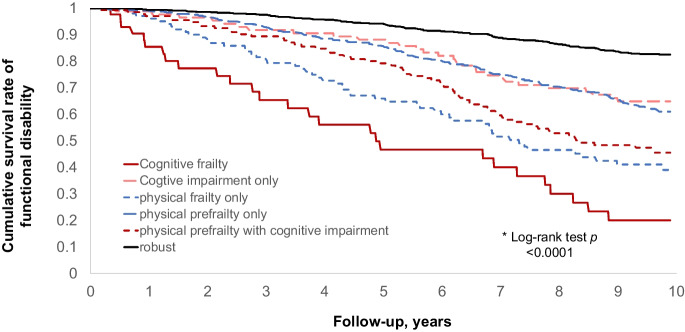
The cumulative survival rate (unadjusted) of functional disability over 10 years according to cognitive frailty status at baseline

The cumulative survival rate (unadjusted) for functional disability by cognitive frailty status at baseline is shown in Fig. 1. Table 3 shows that participants with cognitive frailty had a significantly increased incidence rate of functional disability, with a crude incidence rate of 146.7/1000 person-years in the cognitive frailty group versus 19.0/1000 person-years in the robust group (crude HR 9.29, 95% CI 6.08‒14.19, *p* < 0.001). Compared with being both physically and cognitively robust, the multivariable-adjusted HRs (95% CIs) for functional disability were 3.70 (2.37‒5.77) for cognitive frailty, 2.51 (1.81‒3.47) for physical pre-frailty with cognitive impairment, 2.16 (1.42‒3.29) for cognitive impairment only, 1.95 (1.36‒2.80) for physical frailty only, and 1.94 (1.53‒2.46) for physical pre-frailty only. The multivariable-adjusted HR (95% CI) for the cognitive frailty group was 1.71 (1.00‒2.95) compared with the cognitive impairment only group, and 1.89 (1.18‒3.03) compared with the physical frailty only group. The Fine and Gray model analysis showed that the association between cognitive frailty and functional disability changed minimally when adjusted for the competing risk for death (Table 4). The association between cognitive frailty and risk for functional disability did not vary by sex or age groups (all *p*-values for interactions > 0.05).

**Table 3:** Association between cognitive frailty status and the risk of functional disability over 10 years (N = 1,597)

	No. of events/ participants	Incidence rate per 1000 person-years	Crude model	Age- and sex- adjusted model	Multivariable-adjusted model^*^
			HR (95%CI)	HR (95%CI)	HR (95% CI)
Cognitive frailty status					
Robust	108/657	19.0	1.00 (reference)	1.00 (reference)	1.00 (reference)
Pre-frailty only	204/568	46.4	2.54 (2.01‒3.20)	1.96 (1.55‒2.48)	1.94 (1.53‒2.46)
Frailty only	53/103	88.5	5.24 (3.77‒7.29)	2.11 (1.48‒3.00)	1.95 (1.36‒2.80)
Cognitive impairment only	29/86	42.2	2.29 (1.52‒3.44)	2.03 (1.34‒3.06)	2.16 (1.42‒3.29)
Pre-frailty and cognitive impairment	67/137	72.1	4.10 (3.02‒5.57)	2.53 (1.84‒3.46)	2.51 (1.81‒3.47)
Cognitive frailty	27/46	146.7	9.29 (6.08‒14.19)	4.15 (2.68‒6.44)	3.70 (2.37‒5.77)

Note: CI = confidence interval; HR = hazard ratio

^*^Multivariable-adjusted model adjusted for age (years, continuous), sex (men or women), education (years, continuous), economic status (very uncomfortable/uncomfortable or comfortable/very comfortable), currently employed (yes or no), living alone (yes or no), smoking (yes or no), drinking (yes or no), exercise habit (yes or no), body mass index (kg/m^2^, continuous), history of falls (yes or no), multimorbidity (yes or no), history of hypertension (yes or no), stroke (yes or no), chronic heart disease (yes or no), and diabetes mellitus (yes or no)

**Table 4:** Association between cognitive frailty status and the risk for functional disability over 10 years with adjustment for competing risk for death (N = 1,597)

	Crude model	Age- and sex- adjusted model	Multivariable-adjusted model^*^
	HR (95% CI)	HR (95% CI)	HR (95% CI)
Cognitive frailty status			
Robust	1.00 (reference)	1.00 (reference)	1.00 (reference)
Physical pre-frailty only	1.98 (1.37‒2.88)	1.79 (1.46‒2.19)	1.77 (1.43‒2.18)
Physical frailty only	4.79 (3.56‒6.44)	2.00 (1.42‒2.82)	1.93 (1.38‒2.70)
Cognitive impairment only	1.98 (1.37‒2.88)	1.71 (1.18‒2.49)	1.79 (1.22‒2.63)
Physical pre-frailty and cognitive impairment	3.55 (2.72‒4.64)	2.20 (1.63‒2.96)	2.20 (1.62‒3.00)
Cognitive frailty	9.49 (6.20‒14.53)	4.22 (2.77‒6.42)	3.91 (2.56‒5.97)

Note: CI = confidence interval; HR = hazard ratio

^*^Multivariable-adjusted model adjusted for age (years, continuous), sex (men or women), education (years, continuous), economic status (very uncomfortable/uncomfortable or comfortable/very comfortable), currently employed (yes or no), living alone (yes or no), smoking (yes or no), drinking (yes or no), exercise habit (yes or no), body mass index (kg/m^2^, continuous), history of falls (yes or no), multimorbidity (yes or no), history of hypertension (yes or no), stroke (yes or no), chronic heart disease (yes or no), and diabetes mellitus (yes or no)

In the sensitivity analyses, the association between cognitive frailty and functional disability persisted after adjusting for potential reserve causality by excluding participants with incident functional disability in the first 2 years of follow-up (Supplemental Table 2) and those aged ≥ 85 years (Supplemental Table 3). Excluding participants with a self-reported history of depression (Supplemental Table 4) and those with MMSE scores < 21 (Supplemental Table 5) did not materially change the results.

## Discussion

In this prospective cohort study involving older Japanese adults, cognitive frailty was associated with an approximately four-fold increase in the risk for functional disability compared with robust individuals. This finding remained robust after adjusting for the competing risk for death and potential reverse causation. To the best of our knowledge, this is the first 10-year prospective cohort study that addressed the association between cognitive frailty and the risk for functional disability.

Our results were consistent with previous prospective studies in which cognitive frailty was associated with a significantly increased odds ratio for functional disability [21–24]. In the Three-City Study, participants who were frail with cognitive impairment had nearly four times higher odds of mobility disability, over three times higher odds of IADL disability, and 5.6 times higher odds of ADL disability compared with those who were non-frail and without cognitive impairment [24]. A 4-year longitudinal analysis in the China Health and Retirement Longitudinal Study reported the odds of IADL disability were 3.4 times higher among participants with cognitive frailty at baseline compared with those who were robust [21]. Findings from the Singapore Longitudinal Ageing Study showed that pre-frailty/frailty with cognitive impairment was associated with a two-fold increased risk for functional ADL and IADL disability during a 3-year follow-up among adults aged ≥ 55 years [22]. A 5-year cohort study from Malaysia observed a five-times higher risk for functional disability in relation to cognitive frailty at baseline [23]. A 2-year Japanese cohort study showed cognitive frailty was associated with nearly a four-fold higher HR for functional disability based on LTCI certification [25]. In this study, we used time-to-event data over a 10-year follow-up period and demonstrated that cognitive frailty at baseline was associated with an approximate four-fold increase in the risk for functional disability, even after adjusting for potential reverse causation and competing risk for death. In agreement with previous studies [13, 15], we observed a significant interaction of cognitive impairment with physical frailty on the risk of functional disability, supporting the integration of cognitive impairment and physical frailty into the concept of cognitive frailty. In addition, our observation of lower cognitive function in the cognitive frailty group compared to the cognitive impairment-only group may also explain its higher risk of functional disability. Our results, together with previous studies, suggest that cognitive frailty is clinically important and may serve as a target for preventing functional disability in the community setting.

Both physical frailty and cognitive impairment are well-recognized risk factors for dependency among older adults [13]. A population-based brain magnetic resonance imaging (MRI) study showed that cognitive frailty had unique clinical and MRI features compared with normal controls, such as higher levels of white matter hypointensity, periventricular hyperintensity grades, deep and subcortical white matter hyperintensity, higher prevalence of lacunar infarcts and microbleeds, and reduced medial temporal lobe volume [10]. The mechanisms of cognitive frailty, such as accumulation of neurotoxic beta-amyloid in the brain, nigral neuronal loss, cardiovascular factors [30], and malnutrition and depression [30], can also contribute to explaining the strong association between cognitive frailty and functional dependency [13]. Taken together, it seems biologically plausible that cognitive frailty serves as an important risk factor for functional disability.

The major strengths of our study included its prospective cohort design, large population-based sample, long follow-up period, definite follow-up of all participants (unless participants moved away), and the objective measure of physical activity component of physical frailty. However, several limitations should be noted. First, despite excluding dementia as a criterion for cognitive frailty, we could not rule out the possibility of undiagnosed or probable dementia. Sensitivity analysis excluding participants with MMSE scores < 21 showed similar results, indicating this did not alter our conclusions. Second, since older individuals must contact municipal offices to have their functional disability levels officially certified, some frail individuals might not have reported disabilities [43]. This could have resulted in an underestimation of the incidence of functional disability and the strength of the association with cognitive frailty. Third, cognitive frailty and covariates were only measured at baseline, without considering changes during follow-up. This might have biased our results toward the null. Fourth, reverse causation may be likely because of potential uncertified functional disability at baseline. However, sensitivity analyses excluding participants certified with functional disability in the first two years of follow-up and those aged ≥ 85 years showed minimal changes in results. Fifth, participants included in the final sample may have been more physically active and healthier than the general population, because of excluded participants who had inadequate accelerometer wear time and missing data on covariates. This may have caused a selection bias, resulting in an underestimation of the prevalence of cognitive frailty at baseline and the strength of the association between cognitive frailty and the incidence of functional disability. Sixth, despite our measurement and adjustment strategies, the possibility of uncontrolled confounding factors, such as silent cerebral ischemia, or residual confounding—such as that arising from self-reported medical histories of chronic diseases—cannot be ruled out. Lastly, this study was conducted in a single Japanese town, which may limit the generalizability of the findings. Caution is advised when generalizing these findings to the wider population of community-dwelling older adults.

## Conclusions

This study demonstrated that cognitive frailty is associated with an increased risk for functional disability in community-dwelling Japanese older adults. This finding underscores the clinical importance of addressing cognitive frailty in the prevention of functional disability among this population. Based on our findings, we encourage clinical trials to explore preventive strategies for cognitive frailty, such as cognitive and motor dual task approaches, to protect against functional disability in older people.

## Supplementary Information

Below is the link to the electronic supplementary material.

Supplementary file1 (DOCX 46 KB)

## Data Availability

The datasets used and/or analysed during the present study are available from the corresponding author on reasonable request.
