# Correction to: Cognitive frailty and functional disability in older adults: A 10‑year prospective cohort study in Japan

**DOI:** 10.1007/s11357-025-01618-5

**Published:** 2025-03-28

**Authors:** Sanmei Chen, Tao Chen, Takanori Honda, Hiro Kishimoto, Yu Nofuji, Kenji Narazaki

**Affiliations:** 1 Graduate School of Biomedical and Health Sciences, Hiroshima University, Minami Ward, 1‑2‑3 Kasumi, Hiroshima, 734‑8551 Japan; 2 Sports and Health Research Center, Department of Physical Education, Tongji University, 1239 Siping Road, Shanghai, 200‑092 China; 3 Center for Cohort Studies, Graduate School of Medical Sciences, Kyushu University, 3‑1‑1 Maidashi, Higashi‑Ku, Fukuoka, 812‑8582 Japan; 4 Faculty of Arts and Science, Kyushu University, 744 Motooka Nishi‑Ku, Fukuoka, 819‑0395 Japan; 5 Research Team for Social Participation and Healthy Aging, Tokyo Metropolitan Institute for Geriatrics and Gerontology, 35‑2 Sakae, Itabashi, Tokyo 173‑0015 Japan; 6 Center for Liberal Arts, Fukuoka Institute of Technology, 3‑30‑1 Wajiro‑Higashi, Higashi‑Ku, Fukuoka, 811‑0295 Japan

**Correction to: GeroScience**

10.1007/s11357-024-01461-0

The article Cognitive frailty and functional disability in older adults: A 10‑year prospective cohort study in Japan, written by Sanmei Chen, Tao Chen, Takanori Honda, Hiro Kishimoto, Yu Nofuji and Kenji Narazaki, was originally published under exclusive license to © The Author(s), under exclusive licence to American Aging Association 2024. As a result of the subsequent decision to publish the article under the open access model, the article’s copyright notice was changed on March 5, 2025 to © The Author(s), 2025 and the article is now distributed under a Creative Commons Attribution-NonCommercial-NoDerivatives 4.0 International License, which permits any non-commercial use, sharing, distribution and reproduction in any medium or format, as long as you give appropriate credit to the original author(s) and the source, provide a link to the Creative Commons licence, and indicate if you modified the licensed material. You do not have permission under this licence to share adapted material derived from this article or parts of it. The images or othe third party material in this article are included in the article’s Creative Commons licence, unless indicated otherwise in a credit line to the material. If material is not included in the article’s Creative Commons licence and your intended use is not permitted by statutory regulation or exceeds the permitted use, you will need to obtain permission directly from the copyright holder. To view a copy of this licence, visit http://creativecommons.org/licenses/by-nc-nd/4.0/.

