# Supplementary material for: Cognitive frailty and functional disability in older adults: A 10-year prospective cohort study in Japan
Source: GeroScience. 2024 Dec 3;47(3):5057–67. doi: 10.1007/s11357-024-01461-0 (PMC12181443; doi:10.1007/s11357-024-01461-0)
Supplement: Supplementary file 1 — Supplementary file1 (DOCX 46 KB) [file 11357_2024_1461_MOESM1_ESM.docx]

Excluded:

n = 780 Incomplete data on measures of physical frailty

n = 144 incomplete data on MMSE

n = 72 Missing data on covariates

Excluded:

n = 9 being identified as requiring LTCI before the date of their baseline assessment

n = 10 Having a medical history of dementia

n = 5 Having a medical history of Parkinson’s disease

n = 12 MMSE <18 points (probable dementia)

Baseline

n = 2,629

Final sample

n =1,597

Eligible sample

n =2,593

Supplemental Figure 1. Flowchart of the sample

| **Supplemental Table 1.** Characteristics of included participants versus excluded participants from the eligible sample (N = 2,593) | | | | |
| --- | --- | --- | --- | --- |
|  | No. of participants  with missing data | Included  (n = 1,597) | Excluded  (n = 996) | *p* value^†^ |
| Men, n (%) | 0 | 625 (39.1) | 504 (50.6) | <0.0001 |
| Age, years | 0 | 73.4 ± 6.0 | 73.5 ± 6.4 | 0.42 |
| Education, years | 41 | 11.1 ± 2.5 | 11.1 ± 2.6 | 0.99 |
| Economic status, very uncomfortable  or uncomfortable, n (%) | 98 | 634 (39.7) | 310 (34.5) | 0.01 |
| Currently employed, yes, n (%) | 35 | 275 (17.2) | 245 (25.5) | <0.0001 |
| Living alone, yes, n (%) | 24 | 214 (13.4) | 103 (10.6) | 0.04 |
| Currently smoking, yes, n (%) | 41 | 124 (7.8) | 128 (13.4) | <0.0001 |
| Currently drinking, yes, n (%) | 34 | 626 (39.2) | 430 (44.7) | 0.006 |
| Exercise habit, yes, n (%) | 31 | 982 (61.5) | 526 (54.5) | 0.0005 |
| BMI, kg/m^2^ | 674 | 23.2 ± 3.2 | 23.2 ± 3.5 | 0.78 |
| Having a history of fall, n (%) | 37 | 315 (19.7) | 217 (22.6) | 0.08 |
| Multimorbidity, yes, n (%)^*^ | 19 | 756 (47.3) | 414 (42.4) | 0.01 |
| Physical frailty, yes, n (%) | 144 | 149 (9.3) | 7 (9.7) | <0.0001 |
| Cognitive impairment, n (%) | 513 | 269 (16.8) | 116 (24.0) | 0.0004 |
| History of hypertension, % | 19 | 611 (38.3) | 386 (39.5) | 0.53 |
| History of stroke, % | 19 | 55 (3.4) | 45 (4.6) | 0.14 |
| History of chronic heart disease, % | 19 | 222 (13.9) | 112 (11.5) | 0.07 |
| History of diabetes mellitus, % | 19 | 212 (13.3) | 127 (13.0) | 0.84 |

Note: Data were presented as means (standard deviations) or proportion. BMI = body mass index.

^*^Multimorbidity was defined as two or more of the listed chronic diseases: hypertension, stroke, chronic heart disease, diabetes mellitus, dyslipidemia, respiratory disease, digestive disease, kidney disease, osteoarthritis or rheumatism, trauma fracture, cancer, ear disease, and eye disease.

^†^Statistical significance based on the Chi-square test or the T-test, as appropriate.

**Supplemental Table 2.** Hazard ratios for the risk of functional disability over 10 years by cognitive frailty status at baseline after excluding participants who were certified as requiring long-term care in the first 2 years of follow-up (N = 1,538)

|  | Crude model | Age- and sex- adjusted  model | Multivariable-adjusted model* |
| --- | --- | --- | --- |
| Cognitive frailty status |  | HR (95%CI) | HR (95% CI) |
| Robust | 1.00 (reference) | 1.00 (reference) | 1.00 (reference) |
| Pre-frailty only | 2.57 (2.01‒3.28) | 2.01 (1.60‒2.53) | 2.00 (1.56‒2.56) |
| Frailty only | 4.83 (3.36‒6.93) | 1.98 (1.34‒2.91) | 1.82 (1.22‒2.71) |
| Cognitive impairment only | 2.26 (1.47‒3.48) | 2.03 (1.31‒3.13) | 2.18 (1.40‒3.40) |
| Pre-frailty and cognitive impairment | 4.01 (2.90‒5.55) | 2.54 (1.82‒3.54) | 2.52 (1.78‒3.57) |
| Cognitive frailty | 7.48 (4.52‒12.37) | 3.64 (2.18‒6.09) | 3.22 (1.91‒5.42) |

Note: CI = confidence interval; HR = hazard ratio.

*Multivariable-adjusted model adjusted for age (years, continuous), sex (men or women), education (years, continuous), economic status (very uncomfortable/uncomfortable or comfortable/very comfortable), currently employed (yes or no), living alone (yes or no), smoking (yes or no), drinking (yes or no), exercise habit (yes or no), body mass index (kg/m^2^, continuous), history of fall (yes or no), multimorbidity (yes or no), history of hypertension (yes or no), stroke (yes or no), chronic heart disease (yes or no), and diabetes mellitus (yes or no).

**Supplemental Table 3.** Hazard ratios for the risk of functional disability over 10 years by cognitive frailty status at baseline after excluding participants who were aged 85 years and older at baseline (N = 1,519)

|  | Crude model | Age- and sex- adjusted  model | Multivariable-adjusted model* |
| --- | --- | --- | --- |
| Cognitive frailty status |  | HR (95%CI) | HR (95% CI) |
| Robust | 1.00 (reference) | 1.00 (reference) | 1.00 (reference) |
| Pre-frailty only | 2.52 (1.99‒3.19) | 1.89 (1.49‒2.41) | 1.95 (1.53‒2.51) |
| Frailty only | 5.22 (3.70‒7.37) | 2.48 (1.74‒3.54) | 2.38 (1.63‒3.46) |
| Cognitive impairment only | 2.26 (1.49‒3.43) | 2.08 (1.37‒3.16) | 2.37 (1.54‒3.66) |
| Pre-frailty and cognitive impairment | 3.98 (2.90‒5.45) | 2.59 (1.88‒3.57) | 2.74 (1.95‒3.86) |
| Cognitive frailty | 6.74 (4.04‒11.27) | 3.53 (2.10‒5.92) | 3.01 (1.74‒5.21) |

Note: CI = confidence interval; HR = hazard ratio.

*Multivariable-adjusted model adjusted for age (years, continuous), sex (men or women), education (years, continuous), economic status (very uncomfortable/uncomfortable or comfortable/very comfortable), currently employed (yes or no), living alone (yes or no), smoking (yes or no), drinking (yes or no), exercise habit (yes or no), body mass index (kg/m^2^, continuous), history of fall (yes or no), multimorbidity (yes or no), history of hypertension (yes or no), stroke (yes or no), chronic heart disease (yes or no), and diabetes mellitus (yes or no).

**Supplemental Table 4.** Hazard ratios for the risk of functional disability over 10 years by cognitive frailty status at baseline after excluding participants with a self-reported history of depression at baseline (N = 1,585)

|  | Crude model | Age- and sex- adjusted  model | Multivariable-adjusted model* |
| --- | --- | --- | --- |
| Cognitive frailty status |  | HR (95%CI) | HR (95% CI) |
| Robust | 1.00 (reference) | 1.00 (reference) | 1.00 (reference) |
| Pre-frailty only | 2.53 (2.00‒5.56) | 1.95 (1.54‒2.47) | 1.93 (1.52‒2.45) |
| Frailty only | 5.16 (3.70‒7.19) | 2.08 (1.46‒2.96) | 1.92 (1.33‒2.76) |
| Cognitive impairment only | 2.32 (1.54‒3.49) | 2.04 (1.35‒3.08) | 2.16 (1.42‒3.29) |
| Pre-frailty and cognitive impairment | 4.09 (3.01‒5.56) | 2.51 (1.83‒3.44) | 2.48 (1.79‒3.44) |
| Cognitive frailty | 9.31 (6.10‒14.21) | 4.17 (2.69‒6.46) | 3.70 (2.37‒5.78) |

Note: CI = confidence interval; HR = hazard ratio.

*Multivariable-adjusted model adjusted for age (years, continuous), sex (men or women), education (years, continuous), economic status (very uncomfortable/uncomfortable or comfortable/very comfortable), currently employed (yes or no), living alone (yes or no), smoking (yes or no), drinking (yes or no), exercise habit (yes or no), body mass index (kg/m^2^, continuous), history of fall (yes or no), multimorbidity (yes or no), history of hypertension (yes or no), stroke (yes or no), chronic heart disease (yes or no), and diabetes mellitus (yes or no).

**Supplemental Table 5.** Hazard ratios for the risk of functional disability over 10 years by cognitive frailty status at baseline after excluding participants with MMSE score of <21 points at baseline (N = 1,584)

|  | Crude model | Age- and sex- adjusted  model | Multivariable-adjusted model* |
| --- | --- | --- | --- |
| Cognitive frailty status |  | HR (95% CI) | HR (95% CI) |
| Robust | 1.00 (reference) | 1.00 (reference) | 1.00 (reference) |
| Pre-frailty only | 2.54 (2.01‒5.45) | 1.96 (1.55‒2.48) | 1.94 (1.53‒2.46) |
| Frailty only | 5.24 (3.77‒7.29) | 2.10 (1.48‒2.99) | 1.95 (1.36‒2.80) |
| Cognitive impairment only | 2.27 (2.50‒3.44) | 2.01 (1.33‒3.06) | 2.15 (1.40‒3.29) |
| Pre-frailty and cognitive impairment | 4.00 (2.93‒5.45) | 2.47 (1.80‒3.40) | 2.48 (1.78‒3.44) |
| Cognitive frailty | 8.76 (5.58‒13.75) | 4.18 (2.63‒6.64) | 3.60 (2.24‒5.78) |

Note: CI = confidence interval; HR = hazard ratio.

*Multivariable-adjusted model adjusted for age (years, continuous), sex (men or women), education (years, continuous), economic status (very uncomfortable/uncomfortable or comfortable/very comfortable), currently employed (yes or no), living alone (yes or no), smoking (yes or no), drinking (yes or no), exercise habit (yes or no), body mass index (kg/m^2^, continuous), history of fall (yes or no), multimorbidity (yes or no), history of hypertension (yes or no), stroke (yes or no), chronic heart disease (yes or no), and diabetes mellitus (yes or no).
